# Infant nutrition and growth: trends and inequalities in four population-based birth cohorts in Pelotas, Brazil, 1982–2015

**DOI:** 10.1093/ije/dyy233

**Published:** 2019-03-18

**Authors:** Helen Gonçalves, Fernando C Barros, Romina Buffarini, Bernardo L Horta, Ana M B Menezes, Aluísio J D Barros, Marlos R Domingues, Cesar G Victora, Pelotas Cohorts Study Group, Alicia Matijasevich, Andrea Dãmaso Bertoldi, Diego G Bassani, Fernando C Wehrmeister, Iná S Santos, Joseph Murray, Luciana Tovo-Rodrigues, Maria Cecilia F Assunção, Mariangela F Silveira, Pedro R C Hallal

**Affiliations:** 1 University of São Paulo, Brazil; 2 Federal University of Pelotas, Brazil; 3 University of Toronto, Canada; 1 Post Graduate Program in Epidemiology, Federal University of Pelotas, Pelotas, Brazil; 2 Post Graduate Program in Health and Behavior, Catholic University of Pelotas, Pelotas, Brazil

**Keywords:** Infant nutrition disorders, birthweight, body height, anthropometry, socioeconomic factors, Cohort studies

## Abstract

**Background:**

Levels of child undernutrition have declined in many middle-income countries, whereas overweight and obesity have increased. We describe time trends in nutritional indicators at age 1 year in the 1982, 1993, 2004 and 2015 Pelotas (Brazil) Birth Cohorts.

**Methods:**

Each study included all children born in the urban area of the city, with over 4 200 births in each cohort. Children were measured at approximately 12 months of age. Anthropometric indicators were calculated according to World Health Organization Growth Standards. Stunting and wasting were defined as <-2 Z scores for length for age and weight for length, and overweight as >2 Z scores for weight for length. Prevalence was stratified by sex, maternal skin colour and family income.

**Results:**

The prevalence of stunting declined by 53% (from 8.3% to 3.9%) from 1982 to 2015. Wasting prevalence remained stable at low levels (1.8% in 1982 and 1.7% in 2015), whereas overweight increased by 88% (6.5% to 12.2%). Undernutrition was more common among boys, those born to mothers with brown or black skin colour and in the poorest quintile of families. Socioeconomic inequalities in undernutrition decreased markedly over time. Overweight was markedly more common among the rich in 1982, but fast increase among the poor eliminated socioeconomic differences by 2015, when all groups showed similar prevalence.

**Conclusions:**

Our results confirm the rapid nutrition transition in Brazil, with marked reduction in levels and inequalities in undernutrition in parallel with a rapid increase in overweight, which became the main nutritional problem for children.

**Key Messages**

- Stunting prevalence declined from 8.3% in 1982 to 3.9% in 2015, a reduction of 53% overall, being faster (70%) in the poorest quintile than among the richest (11%).
- Wasting prevalence was below 2% in all four cohorts (no clear reason for apparent increase in 2015 when it reached 1.7% compared with 0.6% in 1993 and 2004), with a 50% reduction among the poor and children with Black or Brown mothers.
- Boys presented higher prevalence than girls for stunting (40% higher), wasting (30%) and overweight (20%).
- Overweight prevalence increased by 88% between 1982 and 2015, with a particularly fast increase after 2004; over time, the increase in the poorest quintile was equal to 63% compared with 22% among the richest.
- The current prevalence of stunting in Pelotas is over twice times, and that of underweight over five times, higher than the 2.3% that would be expected in a well-nourished population.

## Introduction

Child malnutrition may result from either undernutrition, overweight or micronutrient deficiencies.^1^ Undernutrition in early life has well-known short-term consequences, particularly the higher severity and mortality of infectious diseases,^2^ as well as the long-term impact on human capital including shorter adult height, reduced reproductive capacity, lower intelligence and attained schooling and reduced adult income and productivity.^3^ Childhood undernutrition followed by rapid weight gain in adolescence or adulthood has also been linked to higher risks of non-communicable diseases.^3^ Recently, there has been growing concern with childhood overweight, as it is a strong predictor of excessive weight and of cardiovascular and metabolic diseases in later life.^4–6^

Stunting (height-for age deficit),^6–9^ underweight (weight-for-age deficit) and wasting (weight-for-height deficit) rates are decreasing globally, whereas prevalence of overweight (high weight-for-height) and obesity is increasing.^1^^,^^5^^,^^10^ About 24% of the world’s children under the age of 5 years are stunted, 7.5% are wasted and 6.1% overweight.^9^ The highest prevalences of stunting and wasting are observed in low- and middle-income countries (LMICs). Stunting prevalence is declining in many countries, where overweight is increasing.^9^

Nationally representative estimates of child nutrition are not available for Brazil since 2007. Analyses of national trends showed massive reductions in stunting—from 37% in 1974–75 to 7% in 2006–07—and in underweight.^11^ National surveys carried out up to 2007 do not show an increase in overweight prevalence among under-five children,^11^ but other data sources suggest that prevalence in the 5–19 years age range is increasing rapidly.^5^

The complex interplay of social, economic and political determinants of undernutrition results in substantial inequalities between population subgroups.^1^ LMICs consistently show socioeconomic inequalities in the prevalence of stunting, which is concentrated in the poorest wealth quintile.^1^^,^^12^^,^^13^ In contrast, differences in childhood overweight prevalence between the richest and poorest quintiles are small in most countries.^1^^,^^2^

We report on time trends in the prevalence of stunting, wasting and overweight at the age of 1 year, in four population-based birth cohort studies carried out in the city of Pelotas in Southern Brazil between 1982 and 2015, with special attention to inequalities according to socioeconomic status, maternal skin colour and sex of the child. Our analyses update a previous report published in 2008, which described time trends up to 2004.^12^

## Methods

Four population-based birth cohort studies were carried out in the city of Pelotas in Southern Brazil (approximate current population of 340 000) in 1982, 1993, 2004 and 2015. In each year, all mothers of hospital-delivered newborns, who resided in the urban area of the city, were invited to participate in the studies. Data were collected on 5914; 5245; 4231; and 4275 live births, respectively. Hospital births account for over 99% of all city deliveries, and refusal rates at recruitment were below 2% in the four cohorts. Participants of the four cohorts have been followed up on several occasions since birth, initially at home and since 2009 at a purpose-built research facility. Further details of the methodology are available elsewhere.^14^

All cohorts included visits around the age of 12 months. The 1982 cohort subsample included all infants who were born from January to April 1982 (*n* = 1, 916). In the 1993 cohort, all low birthweight (<2500 g) children plus a 20% systematic sample of all other newborns were included (*n* = 1, 460). In the 2004 and 2015 cohorts, the visits included the full cohorts.^15^^,^^16^

In the 1982, 1993 and 2004 cohorts, supine length was measured using locally built infantometers with 1-mm precision (AHRTAG, London, UK), custom-built for these studies.^13–15^ In the 2015 cohort, length was assessed using a SANNY ES2000 portable anthropometer (SANNY, Brazil) with 5-mm precision.^15^ Weight was evaluated using Salter CMS mechanical scales with 25-kg maximum and 100-g precision (Salter, Tonbridge, UK) in the 1982 and 1993 cohort. Scales were calibrated weekly with standard weights.^17^ In 2004, the mothers were initially weighed using Tanita electronic scales (Tanita, Tokyo, Japan) with a 150-kg maximum and 100-g precision; next, they held the child in their laps and their joint weight was recorded. The child’s weight was calculated in the data analyses phase, as the difference between the two measurements.^15^ In the 2015 cohort, the mother and the child were first weighed together using a TANITA UM80 scales (TANITA, Japan) with 100-g precision; the mother then handed the child to the interviewer, and her weight was measured. The child’s weight was calculated automatically by the scale as the difference between the two weights.^15^ In all four cohorts, children were weighed without any clothes and about 10% of all measurements were repeated by supervisors for quality control purposes.

Length-for-age, weight-for-length and weight-for-age Z*-*scores were calculated according to the World Health Organization Growth Standards (WHO 2006), using Anthro 2005 software [http://www.who.int/childgrowth/software/en/]. Stunting, wasting and underweight were defined as less than -2 standard deviations (SD) or Z scores of length-for-age, weight-for-length and weight-for-age, respectively. Children with Z scores of weight-for-length above +2 were classified as overweight.^18^

Independent variables included the child’s sex, maternal skin colour and family income. Skin colour was observed by the interviewers in 1982 and classified as white or other; in 1993, colour was also observed and classified as white, brown or black. These three categories were also used in 2004 and 2015, but the information was based on self-report by the woman. The classification of skin colour is based on the recommendations of the Brazilian Census Bureau.^19^ Family income was obtained by summing the monthly wages of all family members—defined as all persons living in the household and sharing meals—and later dividing this continuous variable into quintiles. Further information on these variables is available in the initial article of this Supplement.^14^

Chi square tests for heterogeneity were used to compare the prevalence of outcomes between categories of the exposure variable in each cohort, and chi square tests for linear trends were used to assess changes over time, from 1982 to 2015. As measures of health disparity, we used the slope index of inequality and concentration index to assess income-related inequality.^20^ Time trends were assessed through interaction terms between the explanatory variables and the cohort year (fitted as an ordinal variable starting with 1982) using Poisson regression with robust variance with stunting, wasting and overweight as outcomes. When there was no statistical evidence of interaction, pooled prevalence ratios of the outcomes according to explanatory variables, after adjustment for cohort year, were presented.^21^ In case of interaction, we presented prevalence ratios separately for each cohort. Presence of an interaction indicates that the prevalence ratio associated with one of the exposures is changing over time, that is relative inequality is changing.

All analyses using data of the 12-month follow-up of the 1993 cohort were weighted to correct for the oversampling of low birthweight, by assigning a sampling weight of 0.2 to the latter. All the analyses were performed using the software Stata version 12.1.^22^

Ethical approval for studies was not required in Brazil until 1996. In 1982 and 1993, verbal consent was obtained from caregivers. The 2004 study was approved by the Ethics Committee of the School of Medicine and the 2015 study by the School of Physical Education, Federal University of Pelotas, and free and informed consent form was obtained from the mothers in both years.

## Results

**Figure 1.**
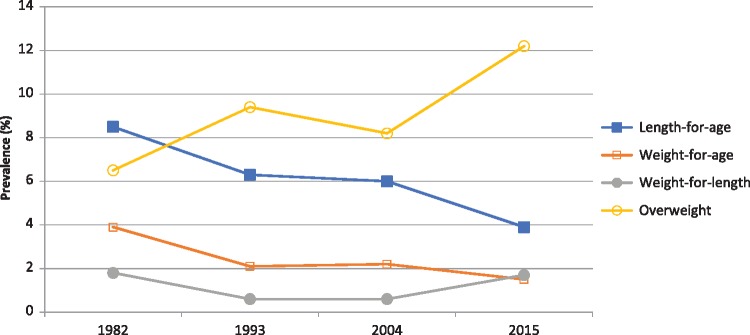
Prevalence of malnutrition (length-for-age, weight-for-age and weight-for-length deficits) and overweight at age 12 months in the 1982, 1993, 2004 and 2015 cohorts.

The proportions of cohort members measured in the 12-month follow-up visits were 79.3% in 1982, 93.4% in 1993, 94.2% in 2004 and 95.4% in 2015. There were important changes in all anthropometric outcomes over the study period (Figure 1).

Stunting prevalence declined from 8.3% in 1982 to 3.9% in 2015, a reduction of 53% (Table 1). In all cohorts, stunting tended to be more common among boys and infants belonging to poor families. Pooled prevalence rates for stunting were 1.4 [95% confidence interval (CI) 1.2; 1.6] higher for boys relative to girls, with no evidence of change over time (*P* = 0.45 for interaction with cohorts). There was also no evidence of a change in prevalence according to maternal skin colour across the four cohorts (*P* = 0.67), with infants born to Black or Brown mothers presenting 1.3 (95% CI 1.1; 1.6) times higher risk than those born to White mothers. The ratio in stunting prevalence between the poorest and richest quintile fell from 5.2 in 1982 to 1.8 in 2015, reflecting the decline of 70% among the poor, compared with only 11% among the rich. There was an interaction between income quintiles and cohort years (*P *= 0.002), which was confirmed by the marked reductions in absolute and relative inequalities over time according to the slope and concentration indices, respectively (Table 2).

**Table 1.** Prevalence of stunting (<-2 Z scores of length-for-age) at age 12 months according sex, maternal skin colour and family income in the 1982, 1993, 2004 and 2015 cohorts

	1982		1993		2004		2015		
	%	95% CI	%	95% CI	%	95% CI	%	95% CI	*P*^a^
Sex	*P* = 0.004^b^		*P* = 0.100^b^		*P* = 0.020^b^		*P* = 0.053^b^		
Males	10.4	8.2; 12.9	7.4	5.5; 9.5	6.8	5.7; 8.0	4.5	3.7; 5.5	<0.001
Females	6.2	4.6; 8.2	5.3	3.9; 7.2	5.1	4.1; 6.2	3.3	2.6; 4.2	<0.001
Maternal skin colour	*P* = 0.133^b^		*P* = 0.027^b^		*P* = 0.062^b^		*P* = 0.245^b^		
White	7.7	6.3; 9.4	5.6	4.4; 7.2	5.4	4.6; 6.3	3.6	2.9; 4.4	<0.001
Brown	10.5^c^	7.1; 15.0	3.9	1.3; 11.5	6.7	4.0; 10.3	5.0	3.3; 7.3	<0.001
Black			10.0	6.6; 14.9	7.7	5.9; 9.8	4.5	2.9; 6.4	
Family income (quintiles)^b^	*P* <0.001^b^		*P* <0.001^b^		*P* <0.001^b^		*P* = 0.012^b^		
Q1 (poorest)	18.7	13.8; 24.5	10.9	7.6; 15.4	8.1	6.3; 10.3	5.6	4.0; 7.5	<0.001
Q2	10.6	7.3; 14.6	8.4	5.8; 12.1	9.1	7.2; 11.4	5.2	3.7; 7.0	<0.001
Q3	6.0	3.6; 9.2	5.5	3.3; 9.1	5.7	4.2; 7.6	3.4	2.3; 5.0	<0.001
Q4	5.5	3.2; 8.7	2.5	1.3; 4.8	3.8	2.6; 5.4	2.4	1.5; 3.6	0.030
Q5 (richest)	3.6	1.8; 6.4	3.2	1.6; 6.5	3.1	2.0; 4.6	3.2	2.0; 4.9	0.767
All children	8.3	6.9; 9.8	6.3	5.1; 7.7	6.0	5.2; 6.8	3.9	3.3; 4.6	<0.001

a *P*-values are displayed from intercohorts chi square test.

b *P*-values are displayed from intracohort chi square test.

c Black and brown were combined.

**Table 2.** Slope index of inequality and concentration index for the outcome variables according to family income quintiles, 1982, 1993, 2004 and 2015 cohorts

Outcome cohort	Slope index of inequality			Concentration index		
	β	SE	*P*	β	SE	*P*
Stunting						
1982	−16.0	2.9	<0.001	−30.5	4.8	<0.001
1993	−14.6	3.0	<0.001	−23.3	4.5	<0.001
2004	−7.6	1.4	<0.001	−21.1	3.4	<0.001
2015	−3.2	1.1	0.004	−13.8	4.7	0.003
Wasting						
1982	−5.6	1.5	<0.001	−50.6	7.8	<0.001
1993	−2.0	1.2	0.085	−31.7	13.9	0.024
2004	−1.5	0.5	0.004	−38.3	10.0	<0.001
2015	−1.0	0.7	0.202	−9.0	7.1	0.204
Overweight						
1982	5.5	2.4	0.023	15.9	5.9	0.007
1993	2.4	2.6	0.351	4.0	5.3	0.451
2004	0.8	2.0	0.587	1.4	3.1	0.643
2015	0.7	1.8	0.716	0.8	2.4	0.738

*P* levels reflect the probability that the index is different from zero (no inequality).

Β, regression coefficient; SE, standard error.

Wasting prevalence was below 2% in the four cohorts (Table 3). There was no interaction between sex of the child and cohort year (*P* = 0.99); the pooled prevalence ratio for boys relative to girls was equal to 1.3 (95% CI 0.9; 1.8) (Supplementary Table 1, available as Supplementary data at *IJE* online). In contrast, there was statistical evidence that ethnic differences in wasting changed over time (*P* = 0.001); the prevalence ratios for black or brown skin colour, relative to white, were 4.6 (95% CI 2.2; 9.9), 3.6 (95% CI 1.0; 12.7), 1.5 (95% CI 0.7; 3.5) and 0.9 (95% CI 0.5; 1.6) in 1982, 1993, 2004 and 2015, respectively. For family income, prevalence was equal to zero in some of the wealthiest quintiles in 1982, 1993 and 2004. For this reason, we grouped the three richest quintiles for analyses and found evidence of an interaction with cohort year (*P* = 0.008). The prevalence ratios among the poorest relative to the three richest quintiles were 6.1 (95% CI 2.3; 15.7), 9.1 (95% CI 2.6; 31.4), 4.5 (95% CI 1.9; 11.1) and 1.5 (95% CI 0.8; 2.6), in the four cohort years, respectively. There was statistical evidence of an interaction with cohort year (Supplementary Table 1, available as Supplementary data at *IJE* online). Accordingly, the slope and concentration indices showed marked declines over time (Table 2). The joint prevalence of stunting and wasting was 0.7% (95% CI 0.2; 1.1) in 1982, 0.3% (95% CI 0.0; 0.6) in 1993 and 0.2% (95% CI 0.0; 0.3) in both 2004 and 2015.

**Table 3.** Prevalence of wasting (<-2 Z scores of weight-for-length) at age 12 months according sex, maternal skin colour and family income in the 1982, 1993, 2004 and 2015 cohorts

	1982		1993		2004		2015		
	%	95% CI	%	95% CI	%	95% CI	%	95% CI	*P*^a^
Sex	*P* = 0.400^b^		*P* = 0.800^b^		*P* = 0.400^b^		*P* = 0.317^b^		
Males	2.1	1.2; 3.5	0.5	0.2; 1.2	0.7	0.4; 0.1	1.8	1.3; 2.5	0.920
Females	1.5	0.7; 2.6	0.7	0.3; 1.6	0.5	0.3; 1.0	1.4	1.0; 2.1	0.934
Maternal skin color	*P* <0.001^b^		*P* = 0.005^b^		*P* = 0.548^b^		*P* = 0.547^b^		
White	1.1	0.6; 1.9	0.4	0.2; 0.8	0.6	0.3; 0.9	1.7	1.3; 2.2	0.041
Brown	5.1^c^	2.7; 8.5	1.0	0.2; 4.1	0.7	0.0; 2.6	1.9	0.9; 3.5	0.001
Black			1.4	0.5; 4.4	0.9	0.4; 1.9	1.2	0.5; 2.4	
Family income (quintiles)^b^	*P* = 0.001^b^		*P* = 0.047^b^		*P* = 0.008^b^		*P*		
Q1 (poorest)	4.6	2.2; 8.2	2.0	0.8; 4.8	1.6	0.8; 2.7	2.1	1.1; 3.4	0.076
Q2	3.0	1.4; 5.6	0.2	0.0; 0.9	0.7	0.2; 1.5	1.5	0.7; 2.6	0.249
Q3	1.6	0.5; 3.7	0.4	0.0; 1.3	0.5	0.1; 1.3	1.5	0.7; 2.6	0.986
Q4	0.7	0.0; 2.3	0.0	–	0.3	0.0; 0.9	1.7	0.9; 2.8	0.026
Q5 (richest)	0.0	–	0.0	–	0.3	0.0; 0.9	1.2	0.6; 2.4	0.010
All children	1.8	1.2; 2.6	0.6	0.3; 1.1	0.6	0.4; 0.9	1.7	1.3; 2.1	<0.001

a p-value are displayed from inter-cohorts chi squared test.

b p-value are displayed from intra-cohort chi-squared test.

c Black and brown were combined.

CI, Confidence interval.

Patterns and trends in underweight prevalence were very similar to those observed for stunting, with a reduction in overall levels and in inequalities related with skin colour and income (Table 4). The summary indices showed important reductions in inequality (Table 2). Overweight prevalence increased by 88% between 1982 and 2015, with a particularly fast upsurge after 2004 (Table 5). There was no statistical evidence of interactions between cohort year and either sex (*P* = 0.17) or skin colour (*P* = 0.10). In the pooled analyses, overweight was 1.2 (95% CI 1.1; 1.4) times more common among boys than girls, and the prevalence ratio for brown and black maternal skin colour relative to white was 0.9 (95% CI 0.8; 1.0) (Supplementary Table 1, available as Supplementary data at *IJE* online). Overweight was more prevalent among children from wealthy than from poor families in 1982 (prevalences of 9.5 in the richest quintile and 4.6 in the poorest quintile, Table 5), but the income gradient disappeared thereafter; there was no evidence of an interaction with cohort year (*P *= 0.43) (Supplementary Table 1, available as Supplementary data at *IJE* online). Over time, prevalence among the poorest showed a 2.7-fold increase, compared with a 1.3-fold increase in the richest quintile. The slope and concentration indices showed marked declines over time, confirming the reduction of inequalities (Table 2).

**Table 4.** Prevalence of underweight (Z score <2 SD for weight-for-age) at age 12 months according sex, maternal skin colour and family income in the 1982, 1993, 2004 and 2015 cohorts

	1982		1993		2004		2015		
	%	95% CI	%	95% CI	%	95% CI	%	95% CI	*P*^a^
Sex	*P* = 0.*7*00^b^		*P* = 0.070^b^		*P* = 0.200^b^		*P* = 0.360^b^		
Males	4.1	2.7; 5.8	2.6	1.7; 4.0	2.5	1.9; 3.3	1.7	1.2; 2.4	<0.001
Females	3.6	2.4; 5.3	1.4	0.9; 2.4	1.9	1.3; 2.6	1.3	0.9; 2.0	<0.001
Maternal skin colour	*P* <0.001^b^		*P* = 0.014^b^				*P* = 0.918^b^		
White	2.9	2.0; 3.9	1.5	0.9; 2.3	1.8	1.4; 2.4	1.6	1.2; 2.1	0.001
Brown	8.6^c^	5.4; 12.7	2.0	0.7; 5.5	1.9	0.6; 4.3	1.6	0.7; 3.0	<0.001
Black			4.5	2.6; 7.8	3.8	2.5; 5.4	1.3	0.6; 2.6	
Family income (quintiles)^b^	*P* <0.001^b^		*P* <0.001^b^		*P* <0.001^b^		*P* = 0.058^b^		
Q1 (poorest)	10.5	6.7; 15.3	5.3	3.2; 8.6	4.5	3.2; 6.2	2.7	1.7; 4.2	<0.001
Q2	5.3	3.0; 8.4	1.8	0.8; 4.0	2.9	1.8; 4.3	1.3	0.6; 2.4	<0.001
Q3	3.8	2.0; 6.5	1.3	0.7; 2.5	1.7	0.9; 2.9	1.2	0.5; 2.3	0.001
Q4	1.3	0.4; 3.0	1.0	0.4; 1.9	0.9	0.3; 1.8	1.2	0.6; 2.2	0.440
Q5 (richest)	0.3	0.0; 1.8	0.0	0.0; 0.9	1.2	0.5; 2.2	1.2	0.5; 2.4	0.102
All children	3.9	2.9; 5.0	2.1	1.5; 2.8	2.2	1.8; 2.7	1.5	3.3; 4.6	<0.001

a *P*-values are displayed from intercohorts chi square test.

b *P*-values are displayed from intracohort chi square test.

c Black and brown colours were combined.

**Table 5.** Prevalence of overweight (>2 Z scores of weight-for-length) at age 12 months according sex, maternal skin colour and family income in the 1982, 1993, 2004 and 2015 cohorts

	1982		1993		2004		2015		
	%	95% CI	%	95% CI	%	95% CI	%	95% CI	*P*^a^
Sex	*P* = 0.005^b^		*P* = 0.100^b^		*P* = 0.400^b^		*P* = 0.016^b^		
Males	8.3	6.4; 10.6	10.7	8.5; 13.7	8.6	7.4; 12.0	13.5	12.0; 15.0	<0.001
Females	4.7	3.3; 6.5	8.1	6.0; 10.6	7.8	6.7; 9.1	10.9	9.5; 12.3	<0.001
Maternal skin colour	*P* = 0.865^b^		*P* = 0.144^b^		*P* = 0.099^b^		*P* = 0.742^b^		
White	6.6	5.2; 8.1	10.0	8.2; 12.2	8.8	7.8; 9.9	12.0	10.8; 13.3	<0.001
Brown	6.3^c^	3.6; 10.0	1.9	2.6; 13.1	5.9	3.4; 9.4	11.9	9.2; 15.0	<0.001
Black			8.7	5.4; 13.9	7.0	5.3; 9.1	13.1	10.5; 16.1	
Family income (quintiles)^b^	*P* = 0.021^b^		*P* = 0.804^b^		*P* = 0.446^b^		*P* = 0.353^b^		
Q1 (poorest)	4.6	2.2; 8.2	9.2	5.9; 14.0	8.4	6.5; 10.6	12.4	10.1; 15.0	<0.001
Q2	6.6	4.1; 10.0	8.0	5.3; 12.1	7.5	5.8; 9.6	10.8	8.7; 13.3	0.027
Q3	3.5	1.8; 6.1	8.9	5.6; 13.8	7.4	5.6; 9.5	13.9	11.5; 16.6	<0.001
Q4	7.8	5.1; 11.4	11.6	7.8; 16.8	9.7	7.8; 12.0	12.5	10.3; 14.9	0.018
Q5 (richest)	9.5	6.5; 13.4	9.4	6.1; 14.3	8.1	6.2; 10.3	12.2	9.8; 15.0	0.148
All children	6.5	5.3; 7.9	9.4	7.8; 11.3	8.2	7.4; 9.2	12.2	11.2; 13.2	<0.001

a *P*-values are displayed from intercohorts chi square test.

b *P*-values are displayed from intracohort chi square test.

c Black and brown colours were combined.

## Discussion

Our results describe how the nutrition transition affected children born in a Brazilian city over a period of more than three decades. Stunting prevalence fell by 53%, from 8.3% in 1982 to 3.9% in 2015, whereas overweight prevalence increased by 88%, from 6.5% to 12.2%. As described in the Introduction, both undernutrition and overweight in early life are important risk factors for a number of conditions along the life course. Our results do not indicate a specific period of time or decade when these changes took place (Figure 1). Taking into account the confidence intervals of the estimates, our results are consistent with steady declines in stunting and underweight, a steady increase in overweight and low, stable levels of wasting. Also, our results do not suggest that the children in Pelotas are facing a double burden of malnutrition, as overweight has replaced stunting, rather than co-existing with stunting, at population level.

The relatively low prevalence of stunting in 1982—less than 10%—must be interpreted in light of the fact that stunting is a cumulative, long-term process that reaches the highest prevalences after 24 months of age.^23^ For example, in the same 1982 cohort, prevalence of stunting was 12.2% at 2 years of age, reaching 25.1% in the poorest group of children.^24^ Globally, it is estimated that approximately 24% of all children under the age of 5 are stunted, and in Latin America the prevalence in this age group was 9.5% in 2016.^9^

Stunting prevalence is declining in many countries.^7^^,^^9^ The reasons behind the decline in stunting in Brazil include improvements in socioeconomic determinants of health, including increased maternal education and poverty reduction, improved coverage with essential services including water supply and sanitation, and universal access to health care.^25^ As shown in previous articles in the present Supplement, time trends in determinants of stunting in Pelotas were consistent with national trends.^14^^,^^19^^,^^26^ In Pelotas as in Brazil as a whole, there were marked reductions in socioeconomic inequalities in stunting, which likely reflect an improvement in living conditions for Brazil’s poor families.^11^

Wasting prevalence remained below 2% in the four cohorts. Low prevalence levels have been observed for many decades in most of Latin America,^27^ where regional prevalence is estimated at 1.3%.^9^ In Pelotas there was an apparent increase in 2015, when prevalence reached 1.7% compared with 0.6% in 1993 and 2004 (Table 3). The reasons behind this increase are unclear. In terms of inequalities, prevalence fell by more than 50% over time for the poorest children and among those born to mothers with black or brown skin colour, whereas children born to white-skinned and upper socioeconomic status mothers presented stable prevalence.

Other articles in this series describe time trends in factors that may have contributed to improved nutrition, including increased income and maternal schooling^14^—particularly for the poor,^14^ fewer teenage pregnancies and lower fertility,^11^ a reduction in maternal undernutrition,^27^ improved health care during pregnancy^28^ and increased breastfeeding duration,^29^ all of which took place in the city during the time period covered by the cohorts.

When interpreting time trends in stunting and wasting, it is important to note that the statistically based prevalence of below -2 Z scores indicates that—even in a population with optimal nutritional conditions—the expected prevalence would be 2.3%.^30^ Both for stunting and wasting, levels in the richest quintile in Pelotas had already been close to this minimum value in 1982, and as a consequence the overall reductions in undernutrition depended solely on improvements among the poor. In 1982, disparities in stunting and wasting presented what is known as a ‘bottom inequality’ pattern, with substantially higher prevalence among the poorest children compared with all other groups.^31^ By 2015, prevalence in the poorest quintile was still higher than for the remaining quintiles, but the differences amounted to a couple of percentage points or less.

In light of Brazil’s success in reducing undernutrition, the main nutritional challenge presented by its children is that of overweight. Childhood body mass index (BMI)—particularly at the age of 2 years or later—tends to track over the life course, with well-described consequences regarding the risk of non-communicable diseases.^3^^,^^32^ Our report of a prevalence of 12.2% among 12-month-old children in 2015 is higher than that of 7.4% estimated for Latin American under-five children.^9^ Although analyses of 5–19-year-olds in Brazil suggested an important increase in overweight prevalence,^6^ paralleling the increase observed among adults,^33^ so far there have been no reports of an epidemic among young children. We report an increase of 88% between 1982 and 2015, with a particularly fast upsurge after 2004; data from national surveys up to 2007 failed to detect such a recent increase.^33^ Causes for the obesity epidemic among Brazilian adults are complex and involve poor diets— and in particular the consumption of highly calorific industrialized foods— as well as reductions in physical activity.^34^ Further studies are required to understand what is driving the obesity epidemic among children. In view of the inverse association between breastfeeding and child obesity,^29^^,^^35^ it will be important to understand the rise in overweight during a period of time when breastfeeding rates have shown substantial increase.^36^

Inequalities in overweight prevalence were reduced. Whereas prevalence was directly associated with family income in 1982, the increase over time was equal to 63% in the poorest and 22% in the richest quintile, thus effectively eliminating inequalities by 2015. Our findings constitute a perverse example where a reduction in inequalities was due to the worsening of nutritional status among the poor.

The official Brazilian classification for ethnicity relies on self-assessed skin colour, a classification that is widely accepted and promoted by Afro-Brazilians, who advocate for disaggregation of government statistics in order to reveal inequities.^19^ Children born to women with black or brown skin colour showed 40% greater risk of stunting when the four cohorts were pooled, with no evidence of a reduction in this ratio over time. In 1982, there were also important ethnic gaps in wasting, but these were no longer present in 2015. Further research is needed to understand why the ethnic gap was reduced for stunting but not for wasting. In contrast, there was no evidence of ethnic differences in overweight prevalence in any of the cohorts. In Brazilian society, ethnicity and socioeconomic status are strongly associated, and this is also the case for Pelotas.^14^ National-level analyses confirm the lower risk of several morbidity and mortality indicators for white-skinned women and their children, compared with those with brown or black skin colour.^37^

Boys had higher prevalence than girls, ranging from 20% to 40% excess, in the three anthropometric indicators studied. It is well-known that the male sex has greater biological frailty in childhood, presenting higher mortality and morbidity rates, and an analysis of 81 countries had previously shown a 14% increase in the risk of stunting among boys than girls.^1^^,^^38^

Our analyses have some limitations. Information on family income was reported by women in the perinatal interview, and may be affected by random error and possibly by systematic error as well, with over-reporting of income by the poor and under-reporting by the rich. In particular, hyperinflation was occurring in Brazil in 1993, and obtaining accurate information on income was problematic. Nevertheless, the clear patterns observed for stunting—which is strongly influenced by socioeconomic conditions^1^—are reassuring. The follow-up rate in the 12-month visit to the 1982 cohort, of 79.1%, was well below those for the other three cohorts, of 93% or higher. This raised the possibility of bias. We investigated this possibility by comparing the prevalence of low birthweight among children who were measured at 12 months (which was 6.8%) and those who were lost to follow-up (7.9%). Follow-up rates showed little variation according to family income (75.6% in the poorest and 82.5% in the highest family income groups), and there were no differences according to sex or skin colour.^39^ These findings suggest that follow-up bias was not important.

Unlike the three more recent cohorts, the visit at 1 year of age to the 1982 cohort was restricted to children born in the first 4 months of the year. The second follow-up to the 1982 cohort (around 2 years of age) included all children born in that year, so that it is possible to compare the prevalence of undernutrition by calendar months of birth.^17^ In this follow-up, the prevalences of stunting, wasting and overweight were 13.3% (95% CI 11.6; 14.9), 0.7% (0.3; 1.1) and 5.4% (4.3; 6.5) for children born January-April, and 14.3% (13.1; 15.5), 0.8% (0.5; 1.1) and 7.7% (6.8; 8.6) for those born May-December. Therefore, there is no evidence of bias for underweight and wasting, and for overweight the prevalence was 2.3% points higher for children born later in the year. Given that the prevalence of overweight in the 2015 cohort was 12.2% (Table 5), even if the results for 1982 were biased downwards, the increase over time is still evident.

The strengths of the studies include their population-based, prospective design, the use of comparable methodology by the same research team over time and—except for 1993—the high rates of follow-up. It should also be noted that Pelotas was one of the six sites providing data for the 2006 World Health Organization Growth Standards,^18^ which were used for assessing nutritional status in the present analyses.

Summing up, our comparison of the four cohorts showed marked improvements in undernutrition over a 33-year period, with concomitant reductions in socioeconomic and, to a lesser extent, in ethnic inequalities. Overweight prevalence, on the other hand, increased markedly, particularly among the poor. The nutrition transition is bringing new challenges to public health in Brazil.

## Funding

The four cohorts received funding from the following agencies: Wellcome Trust, International Development Research Center, World Health Organization, Overseas Development Administration of the United Kingdom, European Union, Brazilian National Support Program for Centers of Excellence (PRONEX), Brazilian National Council for Scientific and Technological Development (CNPq), Science and Technology Department (DECIT) of the Brazilian Ministry of Health, Research Support Foundation of the State of Rio Grande do Sul (FAPERGS), Brazilian Pastorate of the Child and Brazilian Association for Collective Health (ABRASCO).

## Pelotas Cohorts Study Group

Alicia Matijasevich,^1^ Andrea Dãmaso Bertoldi,^2^ Diego G Bassani,^3^ Fernando C Wehrmeister,^2^ Iná S Santos,^2^ Joseph Murray,^2^ Luciana Tovo-Rodrigues,^2^ Maria Cecilia F Assunção,^2^ Mariangela F Silveira^2^ and Pedro R C Hallal.^2^

^1^University of São Paulo, Brazil, ^2^Federal University of Pelotas, Brazil and ^3^University of Toronto, Canada.

**Conflict of interest:** None declared.

## Supplementary Material

Supplementary Tables
