# Supplementary material for: Infant nutrition and growth: trends and inequalities in four population-based birth cohorts in Pelotas, Brazil, 1982–2015
Source: Int J Epidemiol. 2019 Mar 18;48(Suppl 1):i80–8. doi: 10.1093/ije/dyy233 (PMC6422060; doi:10.1093/ije/dyy233)
Supplement: Supplementary Tables [file dyy233_supplementary_tables.docx]

**Supplementary Table 1**. Prevalence ratios for nutritional status indicators at 12 months, according to sex, maternal skin color and family income, showing interactions between these variables and cohort year. Pooled results for the four cohorts.

| **Independent variables** | **Stunting** | |  | **Wasting** | |  | **Overweight** | |  |
| --- | --- | --- | --- | --- | --- | --- | --- | --- | --- |
|  | PR | 95% CI | P interaction | PR | 95% CI | P interaction | PR | 95% CI | P interaction |
| **Sex** (males/females) | 1.39 | 1.19; 1.62 | 0.446 | 1.27 | 0.90; 1.79 | 0.996 | 1.24 | 1.10; 1.40 | 0.171 |
| **Maternal skin color** (brown and black/white) | 1.34 | 1.14; 1.58 | 0.672 | 1.53 | 1.06; 2.21 | 0.001 | 0.90 | 0.78; 1.03 | 0.098 |
| **Family income*** |  |  |  |  |  |  |  |  |  |
| Q1/(Q3-Q5) | 1.94 | 1.62; 2.33 | 0.002 | 2.58 | 1.74; 3.80 | 0.011 | 0.94 | 0.81; 1.10 | 0.283 |
| Q2/(Q3-Q5) | 2.28 | 1.91; 2.73 | 0.239 | 1.46 | 0.93; 2.29 | 0.042 | 0.86 | 0.74; 1.01 | 0.536 |
| * When family income quintiles are fitted as continuous variables, the p levels for interaction with cohort year were equal to 0.001 for stunting, 0.008 for wasting, and 0.425 for overweight. | | | | | | | | | |

**Supplementary Table 2**. Prevalence ratios (PR) for nutritional status indicators at 12 months, according to sex, maternal skin color and family income, for each cohort.

|  | **PR** | **Sex^a^** | **PR** | **Maternal**  **skin color^b^** | **PR** | **Income**  **(Q1x Q3-Q5)^c^** | **PR** | **Income**  **(Q2x Q3-Q5)^c^** |
| --- | --- | --- | --- | --- | --- | --- | --- | --- |
| **Stunting** |  |  |  |  |  |  |  |  |
| 1982 | 1.67 | 1.17; 2.39 | 1.37 | 0.91; 2.06 | 3.7 | 2.50; 5.48 | 2.09 | 1.36; 3.21 |
| 1993 | 1.36 | 0.90; 2.05 | 1.53 | 0.98; 2.41 | 2.93 | 1.77; 4.83 | 2.27 | 1.77; 4.83 |
| 2004 | 1.34 | 1.04; 1.73 | 1.36 | 1.04; 1.78 | 1.92 | 1.41; 2.61 | 2.16 | 1.61; 2.90 |
| 2015 | 1.36 | 0.99; 1.86 | 1.30 | 0.94; 1.80 | 1.73 | 1.20; 2.49 | 1.46 | 1.00; 2.15 |
| **Wasting** |  |  |  |  |  |  |  |  |
| 1982 | 1.42 | 0.66; 3.08 | 4.66 | 2.19; 9.94 | 6.05 | 2.32; 15.73 | 3.94 | 1.47; 10.49 |
| 1993 | 0.73 | 0.21; 2.55 | 3.64 | 1.04; 12.71 | 9.1 | 2.64; 31.41 | 0.94 | 0.18; 4.86 |
| 2004 | 1.40 | 0.63; 3.10 | 1.54 | 0.68; 3.48 | 4.54 | 1.86; 11.08 | 1.91 | 0.63; 5.81 |
| 2015 | 1.28 | 0.79; 2.09 | 0.9 | 0.52; 1.55 | 1.45 | 0.82; 2.56 | 1 | 0.52; 1.92 |
| **Overweight** |  |  |  |  |  |  |  |  |
| 1982 | 1.76 | 1.17; 2.64 | 0.96 | 0.57; 1.61 | 0.66 | 0.35; 1.27 | 0.96 | 0.59; 1.55 |
| 1993 | 1.35 | 0.93; 1.96 | 0.72 | 0.43; 1.19 | 0.92 | 0.56; 1.50 | 0.81 | 0.50; 1.30 |
| 2004 | 1.10 | 0.89; 1.36 | 0.77 | 0.59; 0.99 | 0.99 | 0.76; 1.30 | 0.89 | 0.67; 1.18 |
| 2015 | 1.23 | 1.04; 1.46 | 1.04 | 0.87; 1.25 | 0.98 | 0.79; 1.21 | 0.82 | 0.64; 1.03 |

^a^ females reference

^b^ white reference

^c^ Q3-Q5 reference
